# SHS-Derived Powders by Reactions’ Coupling as Primary Products for Subsequent Consolidation

**DOI:** 10.3390/ma14175117

**Published:** 2021-09-06

**Authors:** Sofiya Aydinyan, Suren Kharatyan, Irina Hussainova

**Affiliations:** 1 Department of Mechanical and Industrial Engineering, Tallinn University of Technology, Ehitajate 5, 19180 Tallinn, Estonia; irina.hussainova@taltech.ee; 2 A.B. Nalbandyan Institute of Chemical Physics, National Academy of Sciences of the Republic of Armenia, P. Sevak 5/2, Yerevan 0014, Armenia; suren@ichph.sci.am

**Keywords:** self-propagating high-temperature synthesis, combustion synthesized powder, thermal coupling, sintering, microstructure, mechanical properties

## Abstract

The capability of self-propagating high-temperature synthesis (SHS) to produce powders that are characterized by a high sintering ability, owing to high heating and cooling rates inherent to the exothermic reaction, is of a special interest for the industry. In particular, SHS-derived powders comprise a significant defect concentration in order to effectively enhance the mass transfer processes during the sintering, which allows for the successful consolidation of difficult-to-sinter materials at relatively low sintering temperatures. From this perspective, the design of precursors suitable for sintering, synthesis in a controlled temperature regime and the optimization of geometrical and structural parameters of SHS powders as a potential feedstock for the consolidation is of key importance. Here, we report on the comparative studies concerning the SHS processing of composites for advanced powder metallurgy techniques. The synthesis and sintering peculiarities of the SHS through coupled reactions in the Me’O_3_(WO_3_,MoO_3_)-Me’’O(CuO,NiO)-Mg-C, Ti-B-Al_12_Mg_17_ systems are comparatively reviewed. The SHS coupling approach was used for the preparation of powders with a tuned degree of fineness (a high specific surface area of particles), a high-homogeneity and a controllable distribution of elements via both the regulation of the thermal regime of combustion in a wide range and the matching of the thermal and kinetic requirements of two interconnected reactions. Microstructural features of the powder feedstock greatly contributed to the subsequent consolidation process.

## 1. Introduction

Self-propagating high-temperature synthesis (SHS) or combustion synthesis (CS) is an exothermic chemical reaction that proceeds in a self-sustaining mode, leading to the formation of valuable solid products. Once initiated by means of a local thermal source, a highly exothermic chemical reaction self-propagates through the heterogeneous medium, converting precursors into products within short reaction times (in the order of seconds or minutes) [1,2,3]. SHS processes are used to synthesize advanced materials, including intermetallics, alloys, pseudoalloys and metal matrix composites, as well as pure metals and refractory ceramics using just high-caloric reacting mixtures, which enable the generation of a sufficient amount of energy. SHS provides a wide range of materials in the form of powders, porous materials, non-porous compacts, casts and coatings. In comparison with the conventional methods of synthesizing materials in high-temperature furnaces, SHS has a number of obvious advantages, namely: a low energy consumption, simple and small-sized equipment, a high productivity and effectiveness, environmental safety, scaling-up ability and the high purity of products due to a self-purification phenomenon at high temperatures. These attractive features endow SHS with a great flexibility, allowing it to both manufacture a wide diversity of products using essentially the same equipment, and establish a number of productions based on them [3]. The most important families of engineering ceramics (metal borides, carbides and nitrides) have also been successfully produced via SHS, both from the elements and from naturally available precursors. Further studies demonstrated the capability of the SHS technique to produce powders that are characterized by a high sintering capacity with respect to powders produced by traditional techniques (furnace, solution methods, etc.) [4,5]. The improved sinterability of SHS powders is attributed to the significant concentration of inborn defects, owing to extremely high heating and cooling processes imminent in the exothermic reaction [5,6,7,8]. These defects are importantly entertained in various physicochemical phenomena, particularly at elevated temperatures. The high concentration of defects in the SHS-derived powders is regarded as being likely to both intensify the mass transfer processes during sintering and allow for the successful consolidation of refractory materials at relatively low sintering temperatures [5,6].

Hence, for a wider application of the SHS method, an innovative direction is developing: the design, synthesis and optimization of SHS powders as a potential feedstock for consolidation via advanced methods, such as additive manufacturing (AM), spark plasma sintering (SPS), hot explosive compaction (HEC), etc.

Spark plasma sintering (SPS) enables the preservation of the microstructural features originated in the combustion wave [9]. It is transpired as an advanced and extraordinary consolidation method to sinter a wide variety of materials that are effortful to sinter by conventional hot pressing techniques. In the SPS, the densification is performed by the application of a pulsed electric field, supplemented with resistance heating and pressure. Recently, the superiority of consolidating materials of a high density in a rather short time and a lower temperature, as compared to conventional sintering techniques, was manifested by the hot explosive consolidation (HEC) method [10,11]. The explosive compaction process of powders is a cost-effective fabrication procedure that is based on the shock wave propagation produced from the detonation of explosives. The shock wave passes through a thin wall of the cylindrical steel container to the powder and consolidates the material due to an induced high pressure. HEC is presumed to be applicable to the fabrication of high-density coupons from SHS-derived ultrafine structured composites.

AM or 3D printing through the selective laser melting (SLM) of metals and selective laser sintering (SLS) of ceramic powders without polymer binders has intrigued the research community, opening a new era of generating the cost-effective and low-waste production of complex shaped, precisely dimensioned and high-value parts [12,13]. The design and development of the advanced SHS technology for AM powders (metals, alloys, ceramics and composites) allow to minimize the manufacturing time and waste of materials and energy, and to facilitate the fabrication of complex parts. The progress of combining SHS and advanced consolidation technologies opens a prospective avenue for the fast, cost-efficient and large-scale production of required powdered materials with a designed architecture. In this regard, SHS-prepared ceramic and composite materials obtained by coupled reactions and compacted by different advanced sintering techniques (HEC, SPS and SLM) are comparatively analyzed below. Here, we report on the design of the SHS route for producing precursors of a controllable microstructure, composition and high purity that are suitable for SPS, HEC or SLM/SLS, which may expand the possibility of SHS to synthesize materials in a controlled temperature regime. From this prospective, the approaches of reactions’ coupling, thermal dilution and mechanical activation become promising [14,15,16]. The reactions’ coupling fuels opportunities for the creation of new energy-efficient processes and broadens the scope of materials that can be synthesized by SHS via the matching of the thermal and kinetic requirements of two interconnected reactions [17,18]. Distinguished advantages of the reactions’ coupling approach in combustion synthesis are (i) the intensification of high exothermic solid-state reactions with strong diffusion retardation or low exothermic reactions; (ii) the enhancement of the concentration limits of combustion; and (iii) the creation of methodologies/strategies that facilitate the design of materials with a required micro(nano)structure and relevant properties, due to the regulation of the thermal regime of combustion in a wide range. The latter aspect is of primary importance for the subsequent consolidation procedure.

SHS processes use only high-caloric reacting mixtures, enabling to generate a sufficient amount of energy, or often, despite being highly exothermic reactions, they cannot proceed in a self-sustaining manner, owing to strong diffusion retardation. On the other hand, reactions with a very high thermal effect are also not suitable for the materials’ synthesis, since they proceed too violently, in a non-controllable explosion mode that leads to the non-complete conversion of reagents, evaporation of precursors and intermediates, agglomeration, etc.

The design of new systems by coupling a low exothermic reaction with a high exothermic one contributes to the temperature-controlled self-sustaining process and to the preparation of suitable powder precursors for the fabrication of counter-bodies. The effective control over the thermal regime of the process enhances the degree of conversion and homogeneity of the combustion product [19,20,21,22].

Another possibility in designing and fabricating novel materials for advanced consolidation with tailored physical-mechanical properties is solution combustion synthesis (SCS) [23], a self-sustained exothermic reaction that takes place in a mixture or homogeneous solution of precursors. Intensive gas evolution during the solution combustion reaction prevents agglomeration and the grain growth of the product at a high reaction temperature. Therewith, the high reaction temperatures will favor the formation of a highly crystalline nanostructure that is beneficial for the sintering processes.

The synthesis and sintering peculiarities of the following SHS-derived composite powders are comparatively reviewed. Among the powders, there are tungsten-copper (HEC), tungsten-nickel (SPS), molybdenum-copper (SPS, SLM) and titanium diboride–AlMgB_14_ (SPS). The preparation of the aforementioned powder feedstock with the tuned degree of fineness (a high specific surface area of particles) of a high-homogeneity and controllable distribution, specific microstructural features and the partial merging of particles by SHS provides a beneficial ground for the successful sintering/densification process and fabrication of bulk samples with enhanced physicomechanical properties.

## 2. The Role of Coupling on the Synthesis and Sintering of SHS Powders

For the implementation of the synthesis of advanced materials in the combustion wave and design of their microstructure, the cause-and-effect relationship between the composition of the reactive mixture-combustion wave structure, propagation velocity-phase composition and microstructure of products is substantial. Great opportunities are offered by the reactions’ coupling approach.

Through the utilization of the coupling approach, the macrokinetic and microkinetic aspects of controlling basic characteristics of the SHS wave-temperature profile, thermal gradients and propagating velocity are tuned in order to obtain the target products of a high purity and desired microstructure.

### 2.1. The Combustion Synthesis by Coupling Approach and Sintering of the Me’-Me’’ Composite Powders

The synthesis and sintering peculiarities of tungsten-copper (HEC), tungsten-nickel (SPS), molybdenum-copper (SPS, SLM) and titanium diboride–AlMgB_14_ (SPS) systems are delivered below.

For the preparation of W-Cu, W-Ni and Mo-Cu composite nanomaterials, the joint magnesio- and carbothermic reduction (using a combined Mg + C reducer) of their corresponding oxides (WO_3_, CuO, NiO and MoO_3_) was successfully designed and accomplished in a guided combustion mode (referred to as a reactions’ thermo-chemical coupling). The use of a Mg + C reducing mixture considers the regulation of the combustion temperature and velocity within a certain range and the implementation of the synthesis in controllable conditions.

**Figure 1:**
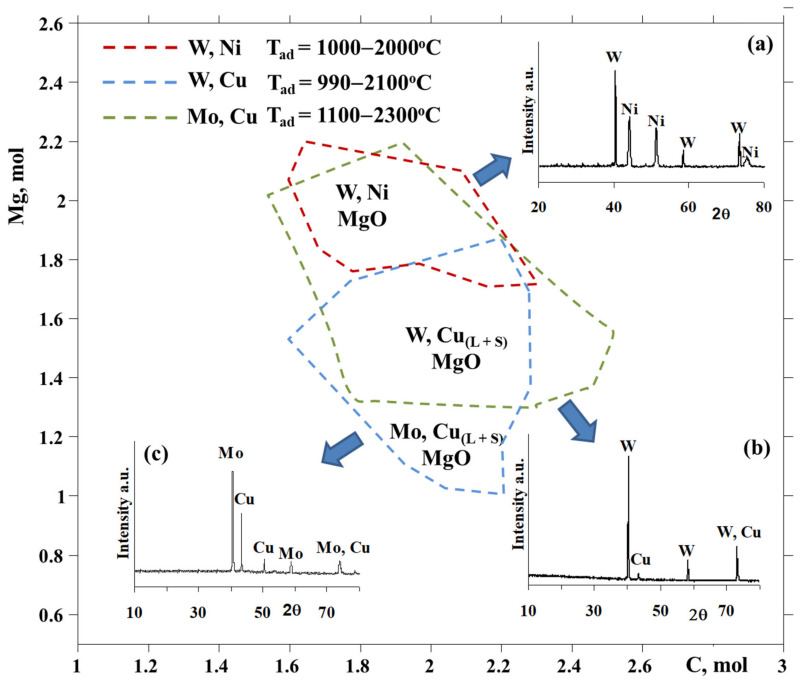
Thermodynamic modelling of WO_3_-CuO-yMg-xC, WO_3_-NiO-yMg-xC and MoO_3_-CuO-yMg-xC systems and XRD patterns of the products (W-Ni (a), W-Cu (b) and Mo-Cu (c)) at optimum conditions, P = 0.3 MPa.

Before the combustion experiments, the thermodynamic calculations (TC) were performed in the quaternary systems in order to synthesize Cu and Ni-refractory metal alloys with 1:1 proportions of metals (Figure 1). The thermodynamic modeling of the quaternary Me’O_3_(WO_3_,MoO_3_)-Me’’O(CuO,NiO)-Mg-C systems showed that the interactions are not a simple summation of corresponding Me’O_3_-Me’’O-Mg & Me’O_3_-Me’’O-C ternary systems. The formation areas and corresponding adiabatic temperatures for Mo-Cu, W-Ni and W-Cu bimetallic systems were calculated depending on the reducers’ amounts (carbon (x) and magnesium (y)). The adiabatic combustion temperature and equilibrium composition of products were deduced and optimized according to the ambient gas pressure. Thermodynamic modeling ensured the use of ≥0.3 MPa ambient gas pressure in order to avoid the evaporation of precursors and intermediates, their mechanical scattering and vigorous gas emissions.

### 2.2. The Essence of Reactions’ Thermal-Kinetic Coupling Approach

**Figure 2:**
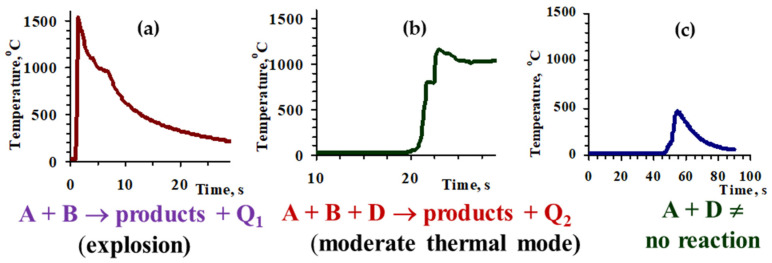
Combustion temperature profiles of the CuO + MoO_3_ + 1.2Mg + xC mixtures, x = 0 (**a**), x = 5.0 (**b**), CuO + MoO_3_ + C (**c**) (Q_1_ and Q_2_ are the amount of heat released). P_N2_ = 0.3 MPa.

**Figure 3:**
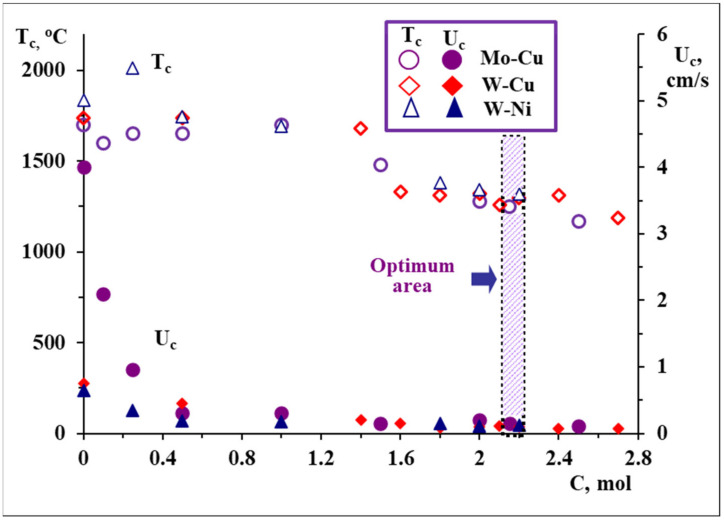
Combustion temperature (T_c_) and combustion velocity (U_c_) vs. carbon content in the WO_3_-CuO-1.3Mg-xC, WO_3_-NiO-1.7Mg-xC and MoO_3_-CuO-1.2Mg-xC systems. P = 0.3 MPa.

The role of coupling on the nature of combustion thermograms is demonstrated on the example of the MoO_3_-CuO-Mg-C system. The combustion process in the (MoO_3_-CuO)(A) + Mg(B) mixture proceeds in an explosion mode (Figure 2a), and the pure carbothermic reaction (MoO_3_-CuO)(A) + C(D) does not have enough exothermicity to self-propagate (Figure 2c), whereas the introduction of carbon to the MoO_3_-CuO-Mg mixture reduces combustion parameters and allows to perform the combustion process at a moderate combustion regime (A + B + D) [24]. Therefore, the addition of carbon has a disproportionate influence on the combustion velocity and temperature, and thereby on the microstructure and phase composition of the products. In particular, the addition of a small amount of carbon (up to 0.7 wt%) to the MoO_3_ + CuO + Mg green mixture causes a decrease in combustion velocity of more than 10-fold (Figure 2a and Figure 3). At that velocity, the combustion temperature decreases by only 300 °C (Figure 3). Note that, in parallel, a sharp decline of the heating rate of the reagents also occur (by one order in the CuO-MoO_3_-Mg-C system) and a double-stage nature of the combustion wave appears on the combustion thermogram (Figure 2b and Figure 3).

According to the XRD analysis of the combustion products, the role of carbon is essential for controlling the reduction degree of metals. For instance, the MoO_3_ + CuO + 1.2Mg + 2.2C composition was found to be optimum for the preparation of the Mo-Cu composite powder (Figure 1c). The coupling of the reactions has a substantial effect on the microstructural characteristics of obtained composite materials; for instance, when increasing the amount of carbon (from 1.4 up to 2.1 mol), a 5-fold decrease in the grain size of the combustion product was observed due to a significant reduction in the combustion temperature [24].

Thus, the role of reactions’ coupling is evident from the phase composition and microstructure features, and from the transformation of combustion thermograms (Figure 2a,b). When carbon is added, the reaction mechanism drastically changes; in particular, the single-stage interaction turns into a double-stage, which establishes favorable conditions for the preparation of target products at a moderate thermal mode.

#### 2.2.1. Copper-Tungsten Bimetallic System

Copper (II) oxide (High grade, STANCHEM, Poland, <40 µm), tungsten (VI) (High grade, Krasniy khimik, Ukraine, <40 µm) oxide, carbon black (P-803, Russia, <1 µm) and magnesium (MPF-3, Russia, 150–300 µm) were used to prepare Cu-W powders in combustion experiments. Cylindrical samples were fabricated from the initial mixture of the reactants via uniaxial pressing (P = 1 kN) and placed in a constant pressure reactor (CPR-3l reaction chamber). The reactor was filled with nitrogen (purity 99.97%) to a pressure of up to 3.0 MPa. The combustion reaction was initiated by a short-term annealing of a tungsten coil. Temperature-time histories of the combustion process were recorded by W-Re thermocouples (5Re/20Re). The preparation of samples for the typical combustion experiment and registration of combustion parameters is thoroughly described elsewhere [1,3,10]. A preliminary thermodynamic consideration of the WO_3_-CuO-yMg-xC system showed that Cu:W of 1:1 composition can be synthesized due to the variation of the amount of Mg between 1.3 and 2.2 mol at a change in carbon amount in the range of 1.5 to 2.5 mol (Figure 1).

The combustion reaction was implemented at a gas pressure of P = 0.3 MPa in order to avoid the evaporation of various WO_x_ oxides, magnesium and copper. According to thermodynamic modelling, the combined magnesio-carbothermic reduction of copper and tungsten oxides was performed in the WO_3_-CuO-yMg-xC system at a fixed amount of Mg (y = 1.3 mol) and various amounts of the carbon reducer. Thermodynamic data were rather supported by experiments and indicated the reasonable choice of reducers’ amounts in order to provide a complete and joint reduction of metals at moderate temperatures (Figure 3) [10].

A number of SHS experiments were performed, with the aim to explore the overall (qualitative and quantitative) impact of carbon on the peculiarities of self-sustained reaction, morphology evolution and phase formation patterns. It was revealed that the decrease in combustion temperature and velocity versus the increase in carbon amount was observed as a result of the increased fraction of low-exothermic carbothermic process (Figure 3).

XRD examinations of the solid combustion products for the WO_3_ + CuO + 1.3Mg + xC mixtures indicated that a carbon amount of 2.1–2.2 mol makes it possible to produce target compounds of combustion (Figure 1b), i.e., W-Cu and its by-product, MgO, which was leached by 10 wt% hydrochloric acid. The preparation of the W-Cu = 1:1 alloy becomes feasible at T_c_ = 1150–1300 °C, which is close to the optimum and low-temperature area of thermodynamic prediction.

**Figure 4:**
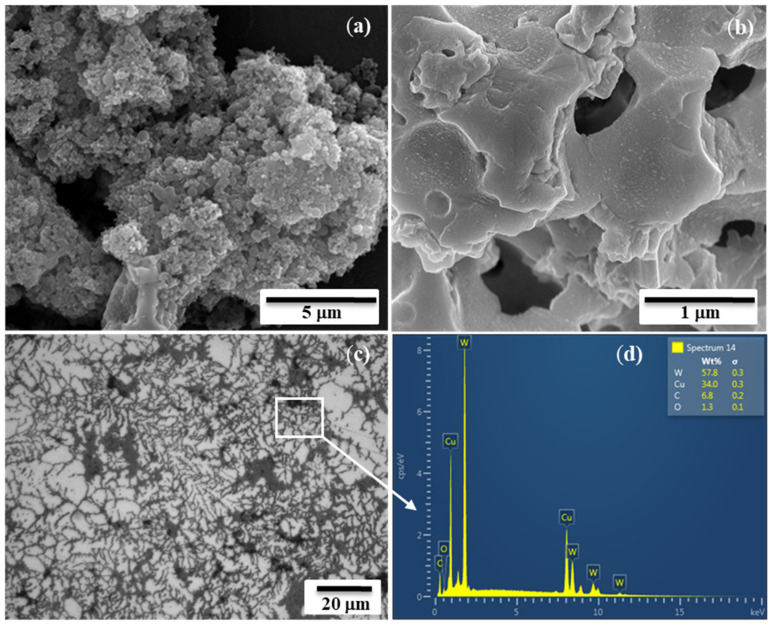
SEM images of SHS-derived W-Cu powder (**a**,**b**) and SEM/EDS of fracture surface of HEC counterparts (**c**,**d**).

Microstructural examinations combined with XRD analysis demonstrated the presence of fine and snowflake-like particles of the W-Cu alloy in the submicron range and with ~1.4 m^2^·g^−1^ SSA (specific surface area) after the acid leaching of the combustion products (Figure 4a). The partial merging of the particulates (up to tenths of microns) in the combustion wave was observed due to a higher combustion temperature than the melting point of copper (Figure 4b). The presence of such agglomerates gives a predisposition for subsequent consolidation via promoting the emergence of intimate bindings among grains and boundaries, thereby reducing diffusion distances and significantly facilitating the sintering of SHS powders.

The fabrication of W-Cu green parts of a high relative density from the combustion synthesized powders was performed via the hot explosive consolidation (HEC) method. For the HEC procedure, the SHS-derived tungsten–copper composite powder was poured into a cylindrical tube container made of steel and pre-densified with a static pressure of 1.5 t load. The compacts of powdered samples were processed by the dynamic pre-densification of coupons at a room temperature and a hot explosive consolidation from 700 to 1050 °C (approximately 10 to 20 K∙s^-1^ within 0.3 to 1 min).

Cylindrical steel tubes of various dimensions were utilized to optimize the consolidation of W-Cu alloys at certain conditions of the detonation pressure, temperature and initial density. Optimized parameters were identified according to pre-densification at room temperature, firstly applying static pressure via compression at different intensities (1.5 t), and then explosive densification at 950, 1000 and 1050 °C, with a loading intensity of 10 GPa [10,11].

HEC-prepared billets from fine SHS-derived powders exhibited a severe diamagnetic susceptibility response. In addition, the microhardness and density measurements manifested that W-Cu alloys obtained by the SHS method via the coupling approach and subsequently compacted by explosive technology comprise an approximately twofold increased microhardness, as compared to those obtained by mechanical alloying (370 kg·mm^−2^ vs 200 kg·mm^−2^), and have a relative density near to the theoretical value (according to both the geometrical and Archimedes values).

The SEM analysis results indicated that 1000 °C is considered an optimum temperature for the complete annealing of microscopic defects and internal stresses from the W-Cu alloys. The optimum annealing temperature was also found to be 1000 °C, according to both the internal friction and Young modulus (E) values. However, they depend not only on the annealing conditions, but also on the history of the sample; in particular, the conditions of powder synthesis, post-treatment, distribution of dendrites, etc.

Microstructural and spectral analyses indicated a homogeneous structure of the obtained W-Cu composites (dark gray corresponds to copper, and light gray corresponds to tungsten) (Figure 4c,d). According to those examinations, the surface of the compacted specimens was almost free of cracks and visible defects. Dendrite formation in W-Cu composite materials is conditioned by the distinct differences in the metals’ melting points, crystal structure and insolubility in each other. The supercooling of the material after HEC and the anisotropy in the surface energy of the tungsten–copper interface led the solid nucleus of tungsten-rich particles to grow in the under-sintered pool of copper in preferred growth directions of the tungsten. Such a highly textured dendrite microstructure of micron-size-W rich dendrites may provide a greater freedom of expansion and contraction (plasticity) when exposed to high thermal loads, suggesting them as the potential candidates for high temperature coatings. On the contrary, the utilization of mechanically alloyed commercial powders for shock wave consolidation has not allowed for the achievement of dense samples (<95%) [25]. High-velocity wave propagation extrudes copper from the adjacent areas of W particles; they instantaneously impact each other and aggregate, creating both pores and the inhomogeneous distribution of constituents.

#### 2.2.2. Nickel-Tungsten Bimetallic System

Nickel (II) oxide (Alfa Aesar, >99%, <44 μm), tungsten (VI) oxide (High grade, Krasniy khimik, Ukraine, <40 µm), carbon black (P-803, Russia, <1 µm) and magnesium (MPF-3, Russia, 150–300 µm) were used to prepare Ni-W powders in combustion experiments. Thermodynamic calculations in the NiO-WO_3_-yMg-xC system demonstrated that the formation of a target product (Ni:W = 1:1 molar ratio) is achievable in a certain amount of carbon (from 1.6 to 2.3 mol) and magnesium (from 1.7 to 2.2 mol) at a 1000–2000 °C adiabatic temperature interval, allowing to design the optimum synthesis circumstances for the simultaneous and entire reduction of tungsten and nickel oxides in a wide range of compositions and thermal conditions (Figure 1) [26]. The probable formation of tungsten carbides (WC and W_2_C) was ruled out during the thermodynamic calculations. According to the results of TC, the amount of magnesium selected was around 1.7–1.8 mol, in order to be sufficient to implement the self-sustaining reaction and provide the complete reduction of oxides at comparatively lower temperatures at a certain amount of carbon. By the next step, the influence of the carbon quantity on the combustion parameters and products’ characteristics was revealed, with the aim to find out the optimum composition of the NiO + WO_3_ + yMg + xC mixture in order to produce the W-Ni alloy (Figure 3). By the same token, the magnesio-carbothermic combined reduction of WO_3_ and NiO was performed via changing the amount of carbon within a prescribed range, in accordance with the TC predicted area. It was revealed that the increase in the carbon amount is responsible for the reduction in the combustion parameters (temperature and velocity), conditioned by a consistent increase in the contribution of the low-caloric carbothermic reaction in the system in one hand, and the change in the interaction mechanism in another [26]. Specifically, at a low amount of carbon, molten NiO participates in the process, and at a high carbon content, solid NiO participates in the process. The investigation of the interaction mechanism in the current mixture revealed that, in contrast to the CuO-WO_3_-Mg-C system, the interaction is initiated with the magnesiothermic reduction of NiO, which is decisive for the heat release and self-propagation of the combustion wave. The reduction process is then continued with the magnesio-carbothermic reduction of WO_3_, and accompanied by the Ni-rich phase (Ni_17_W_3_ or Ni_4_W) formation. An unconsumed amount of oxides reduce up to the metals due to the carbothermic process. In addition, the combustion temperature in the system under consideration is comparatively lower (even lower than the melting temperature of nickel (fusible metal)), and at that temperature, the reduced metallic components are in a solid state and are of more homogeneity as compared to bimetallic systems with molten ingredients. Here, nickel and tungsten form nickel and/or tungsten-rich solid solutions or nickel-rich intermetallic with a particular ratio of elements (Ni_17_W_3_ or Ni_4_W), along with the tungsten-rich phase corresponding to the W-Ni composition (1:1 molar ratio) (Figure 1a).

Based on the XRD patterns of the products, the optimum conditions, according to the initial mixture composition and inert gas pressure, were determined for the preparation of the W-Ni bimetallic system. It was revealed that the conversion degree tends to increase when increasing the amount of carbon in the reactive mixture. The XRD showed that the reduction degree increases with an increase in carbon amount;), the optimum area of the target alloy preparation according to the carbon amount (x = 2.2–3 mol) was also determined. The absence of peaks of metallic nickel and nickel oxide from XRD patterns, as well as the absence of magnetic properties, confirm the formation of the W-Ni bimetallic system. Note that two different combustion modes were observed, according to the carbon amount in the NiO-WO_3_-yMg-xC system: a steady state (with a carbon amount of around 2.2 mol) and an unsteady mode at a high-carbon content (5 mol). The latter corresponds to the spin combustion mode, demonstrating a change in both the microstructure and phase composition of the products [26].

**Figure 5:**
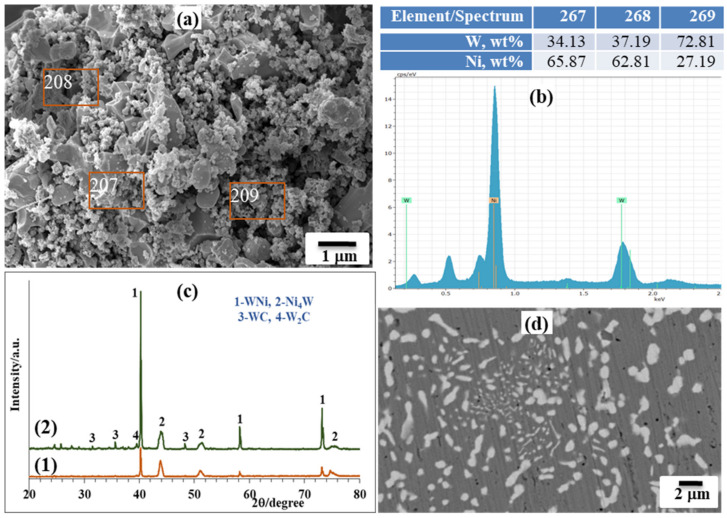
SEM/EDS of SHS-derived W-Ni composite powder after acid leaching (**a**,**b**), XRD pattern (**c**) before (1) and after SPS (2), SEM of surface of SPS-produced bulk sample, 5 min, 1200 °C (**d**).

The target SHS product was crushed into a powder after cooling and hydrochloric acid leaching was used to eliminate byproduct magnesia. XRD and SEM/EDS examinations showed that the product after acid treatment contained mainly Ni and W elements, with an average particle size in the submicron range (Figure 5a,b), illustrating the homogeneous dispersion of both metals throughout the sample. It is obvious that the characteristic peaks of tungsten are broadened and correspond to the W-Ni solid solution, and that the nickel peaks are right-shifted and broadened (Ni-W solid solution).

Emphasizing the influence of reactions’ coupling on the microstructure features, one may observe the fine microstructure formation during the addition of carbon, accompanied with a simultaneous decline in combustion temperature and velocity, and, as a result, the fabrication of the W-Ni bimetallic system at moderate and tailored conditions.

SHS-derived powders were ball milled for 30–60 min at a fixed rotation speed of 200 rpm at a ball-to-powder weight ratio of 4:1, and were subsequently compacted by a spark plasma sintering (SPS) apparatus (KCE^®^-FCT HP D 10-GB, FCT Systeme GmbH, Rauenstein, Germany) in a vacuum (5·10^–2^ mbar). Sintering conditions were as follows: sintering temperature of 950–1350 °C, 50–100 MPa pressure and dwell time of 3–10 min. The heating rate employed was 100 °C·min^−1^ for the ramp-up and the cooling rate was set up to ~200 °C·min^−1^. SPS-produced samples from the Ni-W bimetallic system sintered at 1200 °C temperature and at a 50 MPa pressure within a 5 min dwell time exhibited a 96% relative density.

**Figure 6:**
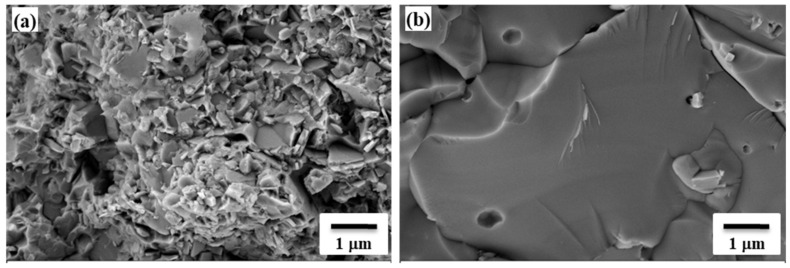
SEM images of fracture surface of SPS produced samples, (**a**) 3 min, (**b**) 10 min, T = 1200 °C.

XRD analysis demonstrated no dramatic change in phase composition after SPS processing (Figure 5c). Trace amounts of tungsten carbides observed are conditioned by the carbon uptake during sintering due to the tungsten’s affinity to carbon at the sintering temperature; as powders were consolidated in graphite dies, graphite punches and protective graphite foil were also utilized. Vickers microhardness was measured to be 5.8 ± 0.6 GPa (HV1), which is comparable (or even higher) with results reported elsewhere [27,28,29] for the Ni-W alloys (4.5–5.5 GPa). The increased microhardness value is solely conditioned by an increased W amount in the Ni lattice structure. The microstructure of the alloy after sintering illustrates the homogeneous distribution of the tungsten-rich phase (light grey) in the nickel-rich phase matrix (dark grey) (Figure 5d). In addition, the influence of the sintering duration showed that 3 min of sintering is not enough to complete the process, as there are under-sintered areas; and 10 min resulted in fusion of the powder (Figure 6).

**Figure 7:**
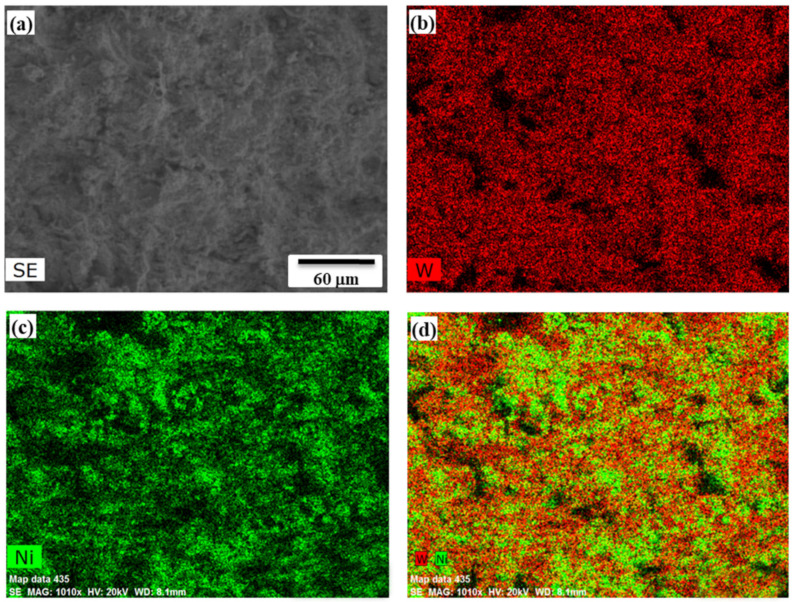
EDS mapping of fracture of the Ni-W SPS-produced sample, 5 min, 1200 °C (**a**), W (**b**), Ni (**c**), W, Ni (**d**).

EDS mapping of the fractured surface revealed the relatively homogeneous distribution of Ni and W elements (Figure 7). An increase in dwelling time of up to 10 min allowed to reduce the porosity to 1%. A further increase in sintering duration is not desirable, as the Ni_2_W_4_C phase may be formed from the surface to throughout the thickness of the sintered compact [30]. However, due to partial agglomeration and the pre-sintered state of the SHS-derived Ni-W powder, at a moderate sintering duration (up to 10 min), the diffusion of carbon occurs only on the surface of powder particles, in contrast to mechanically alloyed powders. In particular, interfaces between grains of initial powders remain structurally different from the volume, offer tracks for quick carbon diffusion and favor the carbides’ formation. In [31], mechanical alloying was used to both in situ fabricate the Ni-W solid solution alloy matrix from a Ni-30 wt% W powder mixture and sinter coupons at 1000 °C during 3 min of a 96–97% relative density. Microstructural examinations showed the homogeneous distribution of phases in a micrometer range; however, the materials were characterized by some residual porosity. The authors suggested to utilize annealing prior to SPS, which may allow eliminating contaminations and increasing the relative density. Another approach to enhance the microstructural homogeneity and relative density of the Ni-W composite is using NiWO_4_ as a precursor for Ni-W alloy synthesis, or avoiding graphite dies during the sintering, which changes the reciprocal diffusion in the sintered material paired with h-BN or aluminum, as suggested in [32].

#### 2.2.3. Copper-Molybdenum Bimetallic System

As Mo-Cu materials with a homogeneous microstructure are difficult to achieve with conventional methods, various attempts have been carried out to produce fine and homogeneously dispersed Mo-Cu powders, utilizing a similar pathway for W-Ni and W-Cu composite materials. The increased scientific intrigue in Mo-Cu materials is conditioned by the fact that Mo and Cu form a pseudo-alloy or composite material, where constituents are either the matrix or dispersing component depending on the ratio of Mo and Cu. In order to achieve the purpose, oxygen-containing compounds of molybdenum and copper were jointly reduced utilizing the reactions’ coupling approach and, in particular, the Mg + C mixture was used as a combined reducer [24]. The real possibility of the joint and the complete reduction of Mo and Cu metals were manifested at a certain amount of reducing mixture (certain ratio of Mg and C) and at moderate propagation velocity of the combustion wave. The obtained powders were consolidated using SPS in a vacuum at a sintering temperature of 950–1050 °C, with the simultaneous exploitation of 100 MPa pressure for a fixed dwell time (3, 5 or 10 min). The mould of 10 mm in diameter was charged with SHS-derived powders and heated up to a defined sintering temperature with a heating rate of 100–200 °C·min^−1^.

Three pathways of powder synthesis were utilized.

**Figure 8:**
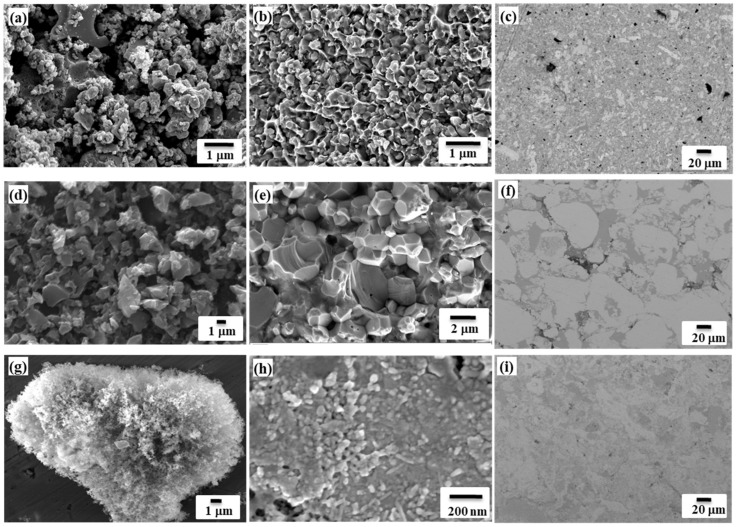
SEM of Mo-Cu powders obtained by different pathways (**a**,**d**,**g**); SEM images after SPS of samples fracture and surface of Cu-Mo obtained from SHS + SCS pathway (**b**,**c**); Cu-Mo obtained from SHS of oxides (**e**,**f**); Cu-Mo obtained from SHS of CuMoO_4_ salt (**h**,**i**); T = 950 °C, P = 100 MPa, t = 6 min, d = 10 mm.

One of the pathways is the preparation of Mo-Cu nanocomposites by the combination of energy-efficient solution combustion and the self-propagation of high-temperature synthesis. In the first step, a MoO_2_ + Cu nanopowder mixture with an average particle size of 50 nm was prepared by SCS using ammonium heptamolybdate, copper nitrate as precursors and citric acid as a chelating reagent. During the second step via the SHS reduction of a fine MoO_2_ + Cu powder mixture by a Mg/C combined reducer, a Mo-Cu nanocomposite (with 50–70 nm size) was produced (T_c_ = 1250 °C) and subjected to SPS at 950 °C for 6 min and 100 MPa pressure. SEM images demonstrate the particle size and shape preservation during the sintering (Figure 8a–c) [33]. The relative density of the samples was 97% and the Vickers hardness was around 3.8 GPa.

Using the second approach, Mo-Cu powders of 1–3 µm particle size were produced by the SHS of the CuO-MoO_3_-yMg-xC mixture (y = 1.2; x = 2.1–2.2 mol; T_c_ = 1300 °C) from copper (II) oxide (High grade, STANCHEM, Poland, <40 µm), molybdenum (VI) (High grade, Pobedit Company, Russia, <15 µm) oxide, carbon black (P-803, Russia, <1 µm) and magnesium (MPF-3, Russia, 150–300 µm) (Figure 3). Powders consolidated at identical conditions are presented in Figure 8d–f. Sintered counter bodies comprise a 4.1 GPa Vickers hardness and a 95% relative density. In contrast to the sample derived by the first pathway, the Mo-Cu compact here has more porosity, but a higher hardness, most likely conditioned by a coarser grain size.

A similar approach was utilized for the preparation of the Mo-Cu bimetallic system from copper molybdates derived by calcination (I) and coprecipitation (II) methods, with a subsequent consolidation [34].

According to the thermodynamic modelling and primary combustion experiments of the copper molybdate reduction using a Mg + C combined reducer, the amount of magnesium was selected as 1.2–1.5 mol in order to achieve complete reduction. The combustion velocity and the combustion temperature tend to slightly decrease with an increase in the carbon content in the mixture with copper molybdate derived by coprecipitation, whereas using calcined CuMoO_4_ caused a substantial drop in the combustion velocity, which was registered by the addition of an even insignificant amount of carbon. The observed pattern was explained by the composition of CuMoO_4_(II) when it was calcined for 1 h, and copper hydroxymolybdate decomposed to copper molybdate during the calcination process and combustion parameters of the calcined CuMoO_4_(II) powder resembled the behavior of copper molybdate(I). With the addition in carbon, the reduction was accomplished by the formation of both metals. Accordingly, the corresponding mixtures with both salts, CuMoO_4_(I) + 1.2Mg + 2.2C and CuMoO_4_(II) + 1.5Mg + 1.6C, were selected for Mo-Cu alloy preparation. Despite the different amounts of reducers in the reactive mixture, both salts followed a similar reduction pathway, firstly converting into salts of Cu_2_MoO_5_ and Cu_6_Mo_5_O_18_, then Cu and MoO_2_ by carbon, and, at the end, into molybdenum by the magnesiothermic reduction of MoO_2_ [34].

XRD examinations of the final products indicate the characteristic diffraction lines of molybdenum and copper after the acid leaching procedure, and the complete elimination of the MgO byproduct. SEM micrographs of the target Mo-Cu products derived from both salts display the existence of spherical nanoparticles (50–100 nm) of a narrow distribution (Figure 8g).

For the consolidation of produced SHS nanopowders Mo-Cu (1:1), the spark plasma sintering (SPS) was utilized. The sintering temperature has a detrimental effect on the density of the produced specimens. Particularly, when the sintering temperature increases by 50 °C (from 950 to 1000 °C), the increase in relative density by 10% occurs. In parallel, a shorter dwelling duration causes grains refinement and, consequently, an increase in hardness. According to multiparametric studies, the optimum sintering temperature was considered to be 1000 °C applied during 3 min for Mo-CuSHS-derived powders. Geometrical and Archimedes densities of Mo-Cu coupons fabricated at optimized conditions were measured as 8.8 g cm^−3^. Accordingly, the relative density was >90%. Microhardness measurements using the Vickers indentation method showed that the HV5 of the Mo-Cu composite makes 3.2 GPa, which exceeds the hardness of pure copper by around ten times and the hardness of molybdenum by more than two times. High-energy ball milling (HEBM) for 1 h certified the increase in hardness of up to 3.8 GPa due to grain refinement; however, the substantial influence of the pre-treatment on the relative density of the compacts was not established. The hardness values for these nanostructured alloys exceeds twice the hardness of the materials prepared by mechanical alloying. The difference is obviously ascribed to the preserved nanostructure at a combination of SHS and SPS techniques (Figure 8h,i).

According to the literature data, Mo-Cu billets sintered without any mechanical pre-treatment comprised spheroidal particles of molybdenum, and the room between them was captured by copper; the maximum density achieved was 88%. A commercial powder mixture of Cu and Mo being subjected to intense HEBM for 1 h led to the formation of a fine structured composite, according to the XRD pattern [35], and Cu and Mo constituents were intermixed at the submicron level; however, SPS at similar sintering conditions did not allow to achieve full density compacts. The comparative analysis of SEM images and the characterization of compacts obtained from SHS-derived powders and HEBM processes demonstrate the privilege of SHS powders by path 1 and path 3 in terms of the microstructural homogeneity of the sintered counterpart and the microhardness. However, for the fabrication of high-density Mo-Cu compacts (98–99%), the laborious and expensive method of the liquid phase sintering of pre-alloyed Mo-Cu nanopowder (100–200 nm) is still preferable [36].

Further, it was proposed to utilize Mo-Cu composite powders with a molar ratio of constituent metals of Mo:Cu = 1:1, obtained from oxide precursors in a combustion mode (Figure 1c and Figure 3), for further densification by the selective laser melting (SLM) technique in order to obtain dense cubic and lattice structured Mo-Cu shapes with the help of the SLM apparatus ReaLizer 50 GmbH (Frankfurt, Germany). Sintering conditions, such as the laser current (mA), scan (mm·s^−1^), point distance (μm) and exposure time (μs), were adjusted for each system under consideration. The SLM machine employs a high-powered continuous-wave laser, which is modulated to function like a pulsed laser system. The Yb:YAG fiber laser, with a maximum power of 120 W and wavelength of 1.07 μm, was used to solidify the structures. The process of SLM was performed in an argon atmosphere of a high purity (99.999 vol.%). Mo-Cu lattice structures showed poor sinterability, and the splashing of the powder and deterioration of the designed structure occurred due to the fine-grained powder. Bulk samples sintered at a 900 mA laser current comprised an 85% relative density. With an increase in the laser current of up to 2500 mA (exposure time 125 µs, point distance 10 µm), a relative density of 93% was achieved, which is comparable with the W-Cu composite obtained by the SLM method (91.6%) [37]. The laser current was revealed to be the most determinative parameter to achieve high relative density compacts of Mo-Cu and W-Cu. Considering the higher laser absorptivity of tungsten and molybdenum compared to copper, it is assumed that refractory metals absorb more laser energy than copper, resulting in the formation of a Cu molten pool and the solid-state sintering of a refractory skeleton almost contemporaneously. Hence, the SLM method has a perspective for the preparation of pseudoalloys comprising two or more distinctly different metals.

#### 2.2.4. Thermochemically Coupled Synthesis of AlMgB_14_-TiB_2_ System with Subsequent Sintering

**Figure 9:**
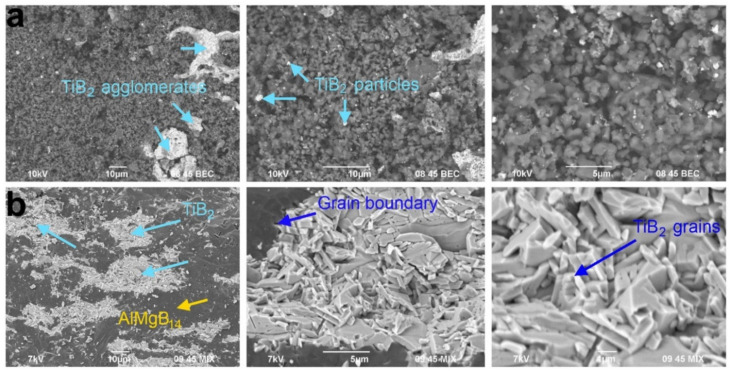
SEM images of SHS powder (**a**) and dense samples sintered by SPS at 1450 °C (**b**). Reprinted from Nikitin et al., 2020 with copyright permission, Ceramics International [38].

The thermochemical coupling approach was used to deliver AlMgB_14_-TiB_2_ composite materials by self-propagating high-temperature synthesis for subsequent SPS compaction at 1470 °C for a duration of 5 min [38]. Ti (OJSC Polema), (purity 99.2%, average particle size 140 μm), Al_12_Mg_17_ (Original powder) (purity 99.2%, average particle size 20 μm) and boron (OJSC Aviabor), (purity 98.8%, average particle size 0.6 μm) were used as precursors in combustion experiments to prepare AlMgB_14_-TiB_2_ composite materials. The powder mixture of Ti and B elements was taken as a donor mixture, and the acceptor mixture encompassed the intermetallic powder of Al_12_Mg_17_ with the amorphous boron powder. For the combustion experiments, (Ti + 2B) and Al_12_Mg_17_:B powder mixtures were mixed in a mass ratio of 70 wt% (Al_12_Mg_17_:B) + 30 wt% (Ti + 2B) in ethanol. Samples with a diameter of 23 mm were cold-pressed from the dried powder mixture. Then, the samples were placed in an SHS reactor. The reactor was evacuated and filled with argon to a pressure of 2.5 MPa. After the reaction initiation in the Ti-B-Al_12_Mg_17_ system, the heating zone was formed enough to melt Al_12_Mg_17_ particles. The melt dissolved the Ti and B elements and the temperature increased up to 1580 °C as a result of the exothermic reaction of the (Ti + 2B) donor mixture. The heat released contributed to the synthesis reaction in the acceptor mixture, with a parallel decrease in the temperature down to 1400 °C. As the donor reaction is exothermic, a further increase in the temperature up to 1550 °C was registered before cooling. Under the influence of the high temperature and with enough time for cooling, TiB_2_ crystal grains grew. The SHS-derived powder material contained particles with a ~1 μm average size (Figure 9). TiB_2_ particles were in the form of agglomerates of up to 30 μm in size due to recrystallization under the influence of elevated temperatures.

The simultaneous consolidation and sintering of the mixture were conducted at a temperature (T_s_) of 1450 and 1470 °C, with 70 MPa of pressure. Heating rates (V_h_) of 50 and 250 °C/min were used during the sintering. After the sintering without holding, the estimated average particle size of both phases, AlMgB_14_ and TiB_2_, was around 3–5 μm. The ceramic structure was not uniform, but was fully dense and exhibited an enhanced hardness of 32.1 GPa. The composite material demonstrated comparable characteristics to the material obtained from the hot pressed (1400 °C, 2 h) mixture of SHS-derived AlMgB_14_ and commercial TiB_2_ [39] in terms of initial powder (particle size and distribution) and final compact (density and hardness).

### 2.3. Discussion of the Results

**Figure 10:**
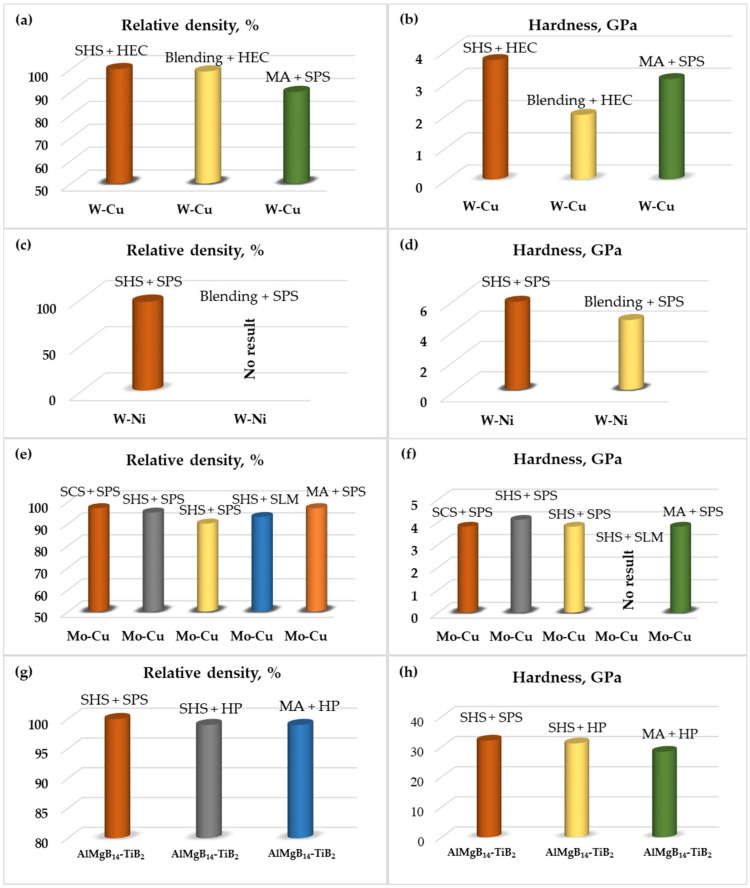
Plots of hardness and relative density values for the systems under consideration; W-Cu (**a**,**b**), W-Ni (**c**,**d**), Mo-Cu (**e**,**f**), AlMgB_14_-TiB_2_ (**g**,**h**).

Comparing the properties of W-Cu composites that were produced by both SHS and blending and consolidated by the same method (HEC), the advantages of SHS-produced powders are obvious in terms of the density and microhardness of the composite, whereas the SPS consolidation of W-Cu nanopowders did not allow to achieve a density >90% [40] (Table 1, Figure 10). However, the particle size of the initial precursor had a strong influence on the hardness value. After SPS, the W-Ni compacts prepared from powders derived by SHS exhibit an enhanced hardness as compared to the compacts produced from the blended precursors. There are no literature data on the consolidation of the Ni-W system via HEC; however, Fe-Ni-W and Al-Ni-W were successfully compacted by HEC [41,42]. Ni-W materials are usually obtained by powder metallurgy methods, such as long-term sintering at a temperature of 1350–1500 °C. In the presence of a liquid phase, the tungsten powder is recrystallized with the formation of almost spherical particles, which are tens of times larger than the particles of the initial powder. The production of the W-Ni powder is possible by SHS at a combustion temperature below the melting point of the constituents, which contributes to the fine powder production. Cu-Mo materials with a homogeneous microstructure are difficult to achieve with conventional methods. Bulks produced from SCS and MA-derived Cu-Mo composite powders demonstrated similar properties after SPS and up to a 97% density. MA is shown to have a beneficial influence on the mechanical properties. Up to now, for the fabrication of high-density Cu-Mo compacts (98–99%), the laborious and expensive method of the liquid phase sintering of the pre-alloyed Mo-Cu nanopowder (100–200 nm) is still preferable. The SLM method is shown to have a perspective for the preparation of pseudoalloys comprising two or more distinctly different metals, but insufficient data are available in the literature so far.

The SHS-derived AlMgB_14–_30%TiB_2_ composite that was compacted by SPS and HP methods demonstrated relatively similar properties: a small difference in hardness could be attributed to a slightly higher (by 70 °C) sintering temperature. The MA-produced composite of a similar particle size and phase distribution exhibited a comparably lower hardness.

## 3. Conclusions

The design of composite systems by coupling a low exothermic reaction with a high exothermic one contributed to the effective control over the thermal regime of the self-sustaining process and to the preparation of applicable powder precursors for the fabrication of counter-bodies. In particular, hot explosively compacted specimens obtained from W-Cu (1:1 molar ratio) fine SHS powders demonstrated an approximately two times enhanced microhardness as compared to those obtained by mechanical alloying. Microstructural observations, in combination with spectral analysis, indicated a homogeneous microstructure evolution of the obtained W-Cu bimetallic system, and a relative density near to the theoretical value. The supercooling of the material after HEC and the anisotropy in the surface energy of the tungsten–copper interface promoted a highly textured dendrite microstructure, which may provide an enhanced plasticity when exposed to high thermal loads, suggesting them as potential candidates for HT coatings.

The fine microstructure formation was observed at the combustion synthesis of Ni-W nanopowder at moderate and tailored conditions. The average value for the Vickers microhardness (5.8 ± 0.6 GPa (HV1)) was achieved as being higher than the values reported elsewhere for the W-Ni alloys (4.5–5.5 GPa), which was as a result of an increased W amount in the Ni lattice structure due to the pre-sintered state of the SHS-derived Ni-W powder. In addition, due to partial agglomeration and the pre-sintered state of the Ni-W powder, at a moderate sintering duration (of up to 10 min), the diffusion of carbon occurs only on the surface of the powder particles, in contrast to mechanically alloyed powders, and contributes to the relative density of the W-Ni composite material.

The comparative analysis of Mo-Cu compacts obtained from SHS-derived powders and HEBM processes demonstrated the privilege of SHS powders obtained from CuMoO_4_ salt and using SCS methods in terms of the microstructural homogeneity of the sintered counterpart and the microhardness. However, for the fabrication of full density Mo-Cu compacts, the liquid phase sintering of pre-alloyed Mo-Cu nanopowder (100–200 nm) is still preferable. However, the perspective of the SLM method for the preparation of Mo-Cu and W-Cu pseudoalloys was successfully demonstrated, owing to the higher laser absorptivity of tungsten and molybdenum compared to copper, assuming a contemporaneous formation of the Cu molten pool and the solid-state sintering of the refractory skeleton. SPS-produced counter-bodies from the thermochemically coupled AlMgB_14_-TiB_2_ system exhibited enhanced mechanical properties. In general, SHS-derived powders obtained by coupled reactions, owing to specific microstructural features, demonstrated themselves as good candidates for subsequent efficient consolidation.

## Figures and Tables

**Table 1:** Comparative overview of the systems under consideration produced by different methods (SHS-self-propagating high-temperature synthesis, SCS-solution combustion synthesis, MA-mechanical alloying, HEC-hot explosive consolidation/compaction, AM-additive manufacturing, HP-hot pressing, SLM-selective laser melting, SPS-spark plasma sintering).

Material *	Particle Size of Powder, Production Method	Sintering Method	Sintering Conditions	Relative Density	Hardness	Refs
W-Cu	From submicron up to tenths of microns, SHS	HEC	1050 °C, pre-densified at 1.5 t	Fully dense	370 kg·mm^−2^	[10]
W-Cu	From nano and micron size elementary powders, blending	HEC	900 °C, 5 GPa	Near full density	126–200 kg·mm^−2^	[11]
W-10 wt% Cu	MA	HEC	950 °C, 3 min	<95	4.34 GPa	[25]
W-Cu	Milling of nanopowders of W (40–60 nm) and Cu (~50 nm)	SPS	950 °C, 10 min	90	3.11 GPa	[40]
Ni-W	Submicron powder, SHS	SPS	1200 °C, 50 MPa, 5 min	96	5.8 ± 0.6 GPa	[26]
Ni-W	Ni and W particles is 14.3 μm and 0.6 μm powder blending	SPS	1000 °C, 30 min	-	486 ± 65 HV	[27]
Mo-Cu	50–70 nm, SCS	SPS	950 °C, 100 MPa, 6 min	97	3.8 GPa	[33]
Mo-Cu	1–3 μm, SHS	SPS	950 °C, 100 MPa, 6 min	95	4.1 GPa	-
Mo-Cu	50–100 nm, SHS	SPS	1000 °C, 3 min	>90	3.8 GPa	[34]
Mo-Cu	1–3 μm, SHS	SLM	2500 mA	93	-	-
Mo-Cu	MA 60 min, precursors 45–100 μm Cu, Mo 2–4.5 μm	SPS	950 °C, 10 min	97	3.68–3.88 GPa	[35]
AlMgB_14-_30%TiB_2_	~1 μm (agglomerates up to 30 μm), SHS	SPS	1470 °C, 5 min	Fully dense	32.1 GPa	[38]
AlMgB_14_-30%TiB_2_	5–10 μm, SHS	HP	1400 °C, 2 h	99	31.2 GPa	[39]
AlMgB_14_-30%TiB_2_	~1 μm, MA	HP	1400 °C, 2 h	99	28.4 GPa	[39]

* Me^’^-Me’’ systems of 1:1 molar ratio were considered.

## Data Availability

The data supporting the findings of this study are available within the article.
